# Validity of 2-point method for load-velocity profiling in free swimming, with snorkel, and with added respiratory dead space mask

**DOI:** 10.1038/s41598-025-23633-4

**Published:** 2025-11-14

**Authors:** Stefan Szczepan, Beata Pożarowszczyk-Kuczko, Kamil Michalik

**Affiliations:** 1 Department of Swimming, Faculty of Physical Education and Sport, Wroclaw University of Health and Sport Sciences, Ignacego Jana Paderewskiego 35, Swimming Pool, Wroclaw, 51-612 Poland; 2 Faculty of Physical Education and Sport, Wroclaw University of Health and Sport Sciences, Wroclaw, Poland

**Keywords:** Physiology, Zoology

## Abstract

This study aimed to evaluate the agreement between the 2- and 3-point models in estimating load-velocity (L-v) profile parameters in swimmers; to compare L-v profile parameters of both models across three respiratory conditions: free breathing (FREE), snorkel (SNOR), and added respiratory dead space mask (ARDS); to examine the relationships between L-v profile parameters (from both models) and a 25 m front crawl performance. Twenty-six male swimmers performed three front crawl semi-tethered 20 m sprints at maximal effort with fixed external loads (1, 4, and 7 kg) under three different respiratory conditions, as well as a 25 m front crawl time trial. The raw data were processed using both 2-point and 3-point models to estimate L-v profile parameters. The two-way ANOVA revealed no significant main effect or interaction in the factors for all L-v parameters. Excellent agreement was observed between the 2- and 3-point models across all conditions for all L-v parameters (*r* ≥ 0.99, R² ≥ 0.98, ICC ≥ 0.99). The 2-point model allows for the assessment of L-v profile parameters as reliably as the 3-point model. The SNOR and ARDS did not affect the L-v profile during swim sprint performance.

## Introduction

Load-velocity (L-v) profiling is a strength-velocity-based training approach originally developed for dry-land training and is commonly used to assess an athlete’s strength, power, and velocity capabilities^1^. To date, the observation of velocity decrements across the L-v spectrum has been increasingly used for monitoring and assessing training programs in many sport disciplines^2^. The L-v approach has also been applied in swimming to assess a swimmer’s strength (propulsion) and velocity capabilities^3^, as well as to determine the optimal loading zone for resistance training^4^. It is also widely recognized as a method that overcomes the limitations of existing equipment-based approaches. Up to now, several indirect methods have been developed to estimate propulsive force, many of which rely on the measurement of active drag (e.g., MAD system, velocity perturbation method, assisted towing methods, etc.)^5–7^. However, directly measuring the complex water flow profile around the swimmer’s entire body, including both propulsive and resistive forces, remains highly challenging for athletes and their coaches^8^. The L-v approach overcomes the limitations of existing methods.

The concept used in strength exercise studies could theoretically be applied to swimming, as swimming velocity and external load demonstrate a similarly strong linear association (R^2^ = 0.98) during the 12.5 m^9^ and 25 m sprint trial front crawl (R^2^ = 0.99)^10^. In swimming practice, semi-tethered swimming has been widely used as part of the L-v testing protocol and is considered a reliable method for this purpose^10^. This method allows for performing similar testing as on-land force-velocity studies^11^. During the test, the swimmer progresses forward through the water, creating relative streamwise water flows around the body with external load applied by a resistance system^12^. Although load and force represent distinct concepts, in swimming, the estimated maximum load is expected to be closely associated with the propulsive forces, since at zero forward velocity, the backward force from the external load must balance the forward force (propulsion) generated by the swimmer’s movement^10^. Hence, the estimation method based on the linear L-v relationship has been adopted to assess indirect propulsive forces with swimming velocity for four swimming strokes: butterfly^3^, freestyle^11^, backstroke^13^, and breaststroke^14^. Furthermore, calculated L-v profile variables such as maximum theoretical load at zero velocity (L_0_), maximum theoretical velocity at zero load (v_0_) can serve to assess, respectively, maximal strength and velocity capabilities during swimming^10^. These parameters showed large and very large correlations (*r* = 0.54 to 0.74) and explained 50 m sprint performance (R^2^ = 0.96)^11^, but there is a dearth of research evaluating this relationship in the 25 m front crawl sprint trial.

Additionally, the steepness of the linear slope for the L-v relationship (slope) can be used as an indicator to explain the factors that determine performance in terms of propulsion and resistance, e.g., how well the swimmer minimizes the resistive force^11^. For instance, a high value of L₀ combined with a low v₀ (a flat slope) means that the swimmer can apply a large force to the water but has a limited ability to use it effectively to produce high swim velocity or is exposed to a large resistive force when moving forward. In contrast, a high value v₀ with a low L₀ can mean that an athlete, despite their weak propulsion, has features that ensure high velocity^11^. Therefore, L-v profiling serves coaches as a practical tool for analyzing and comparing changes in a swimmer’s performance, such as improvements or declines in swimming velocity, as well as for identifying whether these changes are associated with propulsive or resistive forces, and for establishing performance requirements in swimming^14^.

During sprint-based exercises to achieve high swimming velocity, swimmers must not only convert muscular forces into hydrodynamic propulsion^15^, but they also must develop an optimal streamlined position to minimize resistive forces^16^. To help maintain proper head alignment and allow swimmers to focus on acquiring proper technical skills and body position, rather than on breathing, it is common practice to use a center-mount swim snorkel^17^. However, the results show that the time of swim sprint performance was significantly slower when swimming with the special snorkel used for gas exchange measurements, when compared with the free-swimming^18^. Furthermore, the snorkel-imposed changes in the normal biomechanical pattern when swimming front crawl and breaststroke^18^. On the other hand, one of the optional pieces of equipment for swimmers is a specially designed mask with added respiratory dead space (ARDS)^19^, which increases carbon dioxide retention and induces hypercapnia and respiratory acidosis^20^. There is evidence triggering adaptive changes in the circulatory and respiratory systems, as well as in the acid-base balance of the blood after a period of moderate intensity training with ARDS^21^ and high-intensity interval training^22^. Several studies have investigated adaptive changes in lipid metabolism^19^, respiratory muscle strength, and pulmonary function^23^, as well as hematological and immunological status^24^, following several weeks of ARDS practice among swimmers and the use of ARDS warm-up before swim sprint exercise^20^. The physiological effects elicited by using an ARDS mask during maximal sprint efforts may be utilized in training to improve anaerobic metabolism and increase swimming velocity^25^. To date, there is a lack of knowledge about how a swim snorkel and an ARDS mask might induce changes in swimming velocity (especially during maximal anaerobic exercises) and in the L-v profile. It may be beneficial for coaches and researchers to be aware of potential modifications in L-v profile parameters that could result from the use of these types of swim tools during swim sprint performance.

From a technical standpoint, selecting appropriate loads is essential when building L-v profiles. The chosen loads should create a marked reduction in velocity between the lightest and heaviest trials, while ensuring that the heaviest load does not compromise the swimmer’s ability to maintain a stable and consistent stroke pattern^26,27^. In addition, careful consideration should be given to the number of externally loaded trials included in the single testing protocol. In swimming, a multi-point method has been used, ranging from three to twelve points^9^. It was also found that both the 3-point (CV ≤ 2.6%) and 5-point (CV ≤ 3.1%) methods, with increasing loads, were suitable for generating a reliable L-v profile (ICC ≥ 0.90)^10^. However, some limitations of multi-point methods have been noted. Performing sprints across multiple loading conditions can be both time-consuming and physically demanding, potentially resulting in an overestimation of parameters of L-v profile, particularly during heavy-load trials^28^. Moreover, coaches and practitioners may be hesitant to prescribe multiple maximal sprints due to the high physical strain and increased risk of injury. It was found that, for variables with a strong linear correlation, a reasonable approximation can be attained from just two disparate data points, originally referred to as the 2-load method^29^. This was confirmed in the bench press exercise^30^, the bench pull exercise^31^, and in resisted running sprints^32^. Thus, the 2-point method could be an efficient alternative to the standard multiple-point method for L-_V_ profiling^33^. In swimming, the impact of using two loaded trials on the validity of the L-v profile measurement has not yet been established, and this is the first study to investigate this issue in swimming analytically. The linearity of the L-v relationship in swimming and the stability of stroke mechanics across different loads^9^ indicate that two extreme points may be sufficient to represent the L-v profile reliably. Creating a simplified and reliable protocol for assessing individual profiles of L-v may be particularly useful in training and would facilitate broader biomechanical and physiological investigations.

The purpose of this study was threefold. Firstly, to evaluate the agreement between the 2-point model (1 and 7 kg) and the reference 3-point model (1, 4, and 7 kg) in estimating load-velocity profile parameters (L_0_, v_0_, slope) in swimmers. Secondly, to compare L-v profile parameters (L_0_, v_0_, slope) across three respiratory conditions: free breathing (FREE), snorkel (SNOR), and added respiratory dead space (ARDS), using both models. Thirdly, to examine the relationships between L-v profile parameters (from both models) and a 25 m front crawl performance. It was hypothesized that there would be agreement between the 2-point and 3-point models in estimating the L-v relationship, and that removing the middle point (load) from the L-v data would not alter the L-v profile, while their parameters would strongly determine swim sprint performance.

## Methods

### Participants

Twenty-six male swimmers volunteered to participate in this study. All participants were members of the university swimming program and had a World Aquatics score of 448.2 ± 108.2 points. According to the new performance classification model designed to standardize research results in swimming^34^, the participants were classified at the lowest performance level (Level 5, below 449 points). The participants’ characteristics, including anthropometric measurements, competitive swimming experience, and best 50 m freestyle time, are presented in Table 1. Inclusion criteria included having front crawl as the primary competition stroke, at least five years of systematic training, and no injuries or medical conditions at the time of testing, as recommended by the AHA guidelines^35^. Swimmers were excluded if they had sustained an injury or illness within the three months preceding the start of the study. No participants met the exclusion criteria. Swimmers were instructed to maintain their usual lifestyle and diet and to refrain from any additional exercise beyond what was prescribed in the study. All swimmers were reportedly free of drugs, medications, or dietary supplements known to influence physical performance. Participants were informed of the study’s purpose, procedures, and any potential risks. All study participants provided written informed consent following institutional regulations. They were informed of their right to withdraw at any time. No participants withdrew from the study. The study was approved by the Wroclaw University of Health and Sport Sciences Research Ethics Committee (No. 14/2017) and conducted by the Declaration of Helsinki. The minimal sample size *n* = 24 was computed using G*Power 3.1.9.2 software (Franz Faul, Kiel, Germany)^36^ with a large (f^2^ ≥ 0.26) effect size^37^, the level of significance set as α = 0.05, and a power of 1-β = 0.80.

**Table 1:** Swimmers’ characteristics (mean ± SD and 95% CI).

Variables	Mean ± SD	95% CI
Age (years)	22.38 ± 3.76	20.86–23.90
Height (cm)	179.00 ± 7.88	175.82–182.18
Body mass (kg)	77.88 ± 11.96	73.05–82.72
BMI (kg·m^−2^)	24.22 ± 2.62	23.16–25.28
Experience (years)	8.15 ± 4.49	6.34–9.97
50 m Freestyle PB (s)	27.00 ± 2.70	25.91–28.09
50 m Freestyle SB (s)	27.47 ± 2.70	26.38–28.56
World Aquatics Points 2024 (n)	448.17 ± 108.23	404.46–491.89

BMI - body mass index, PB - personal best, SB - season best.

### Procedure

The research was conducted in February, two months after the national championships, during the second macrocycle of the short-course winter season. During this period, the swimmers participated in six swimming sessions per week (totaling nine hours of aquatic workouts and approximately 36 km) and five dryland sessions (about five hours) weekly. One week before testing, participants completed a familiarization session using the same swim devices as in the experiment and swam 1 km of front crawl at low intensity. The purpose of the familiarization was to help participants become accustomed to the conditions used in the study. The testing was conducted in a short-course (25 m) indoor swimming pool under controlled environmental conditions (water temperature: 27 °C, air temperature: 28 °C, relative humidity: 60%, lighting: 600 lx). Participants completed all tests in their standard training swimsuits.

Before the main session, the swimmers completed a standardized warm-up on dry land and in the water. The dry-land warm-up included the following exercises: arm swings (20 reps), walkouts with twists (5 reps), elastic band pull-aparts (15 reps), scapular push-ups (10 reps), scapular pull-ups (10 reps), and squat jumps (15 reps). The in-water warm-up covered a total distance of 500 m and consisted of the following segments: 100 m swim (easy pace); 2 × 100 m swim (kick); 4 × 25 m (15 m at 90% of 50 m race pace, followed by easy pace to the wall), and 100 m easy swim. After the warm-up, participants rested in a seated position for 10 min, under the protocol used in a previous study^38^.

This study employed a randomized crossover design with a single-group approach. The main experimental procedure consisted of three 20 m front crawl semi-tethered sprints performed at maximal effort under the three different respiratory conditions. During the main sessions, each swimmer completed nine trials (3 conditions × 3 loads). Each trial was performed with a different external load, fixed at 1, 4, and 7 kg, applied in ascending order across the three trials. Limiting the number of semi-tethered sprint trials to three does not significantly affect the accuracy of load-velocity profiling^10^.

A previous study involving national-level male swimmers reported excellent reliability in assessing V₀, L₀, and the slope, with intra-class correlation coefficients ≥ 0.90 and coefficients of variation ≤ 2.6% under test-retest conditions using loads in the range 1–9 kg^10^. For the lower-performance-level swimmers, it is important not to use a load that is too heavy, as it could inhibit a consistent stroke pattern. Hence, a maximum load of 7 kg was considered sufficient for the present protocol. The rest interval between trials with different loads was set at 8 min to ensure full recovery of ATP and phosphocreatine (PCr) and allow participants to perform maximally in consecutive trials^39^. The start of each trial was performed from a prone position without a wall push-off. Swimmers were held by the experimenter at the ankles until the start signal, and throughout the entire trial, they swam on the water surface. Swimmers were verbally encouraged before and during the test to maintain maximal effort. Each testing condition was conducted on a separate day, with a minimum of 24 h between sessions to minimize fatigue-related effects.

### Respiratory conditions

**Fig. 1:**
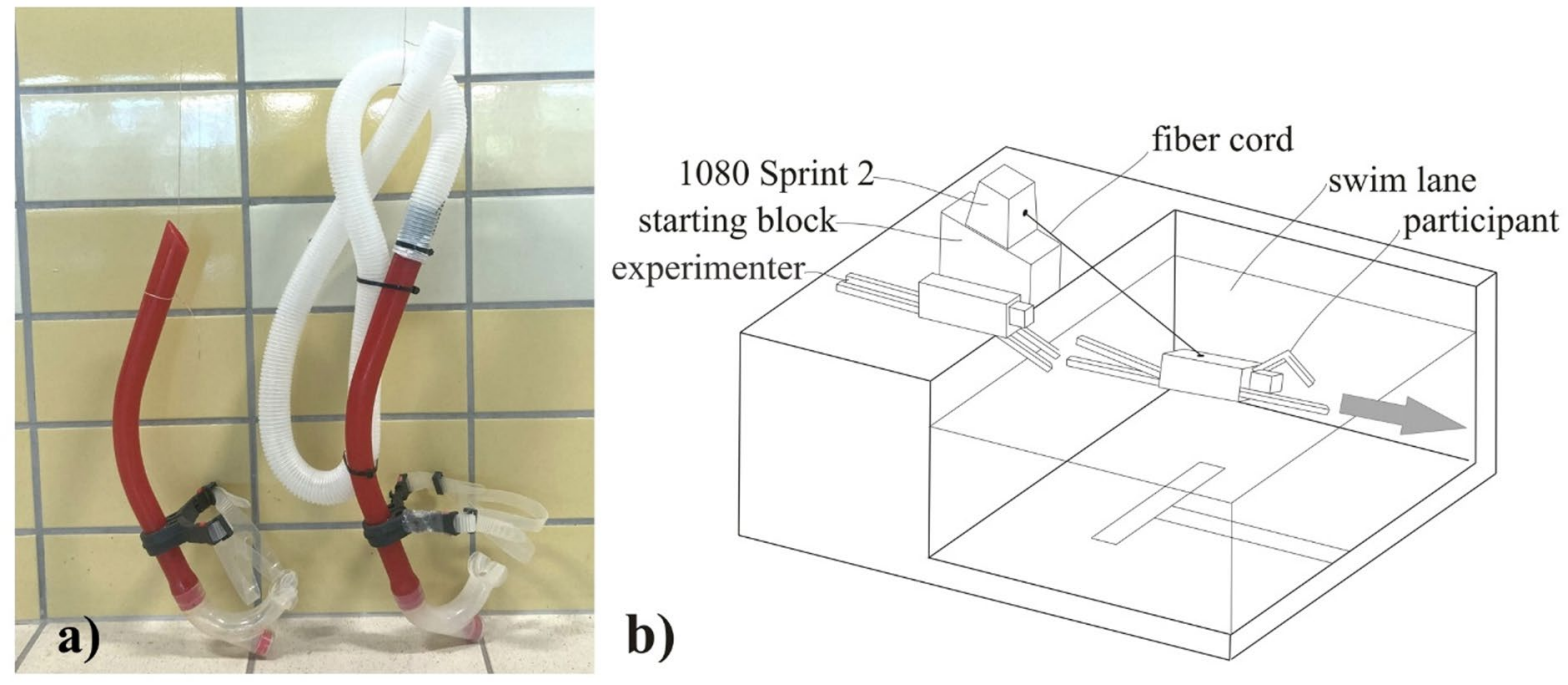
A swim snorkel and ARDS mask were used to induce specific respiratory conditions (**a**). The setup for semi-tethered swimming used to obtain load-velocity profiles (**b**).

Participants were tested under three respiratory conditions: (a) during free swimming without any devices (FREE), (b) with a swim snorkel (SNOR), and (c) wearing an added respiratory dead space mask (ARDS). The swim snorkel used was a standard, front-mounted commercial model (inner diameter: 2 cm, length: 47 cm) (Speedo International Ltd., Nottingham, UK). The ARDS mask was a custom-modified standard swim snorkel (Speedo International Ltd., Nottingham, UK) connected to a ribbed tube with an inner diameter of 3 cm and a total length of 67 cm, designed to increase the anatomical dead space by 1200 ml. The volume of the dead space was verified by filling the tube with water and transferring the contents to a graduated cylinder, as described by Szczepan et al.^19^. The ARDS mask was identical for all participants and rigid enough to maintain a consistent volume during swimming. Previous research included post-exercise individual interviews, and most participants reported no discomfort while using the device^23^. When breathing through either the swim snorkel or the ARDS mask, a nose clip was used to eliminate nasal breathing. In all three respiratory conditions, participants used a free-breathing pattern. A swim snorkel and ARDS mask used to induce specific respiratory conditions have been illustrated in Fig. 1a.

### Load-velocity profiling

A portable resistance device, the 1080 Sprint 2 (1080 Motion, Lidingö, Sweden) was used to provide isotonic resistance. The motor was connected to a composite fiber cord attached to the swimmer’s back via a belt. The device’s integrated software collected velocity data at a sampling rate of 333 Hz. The device was mounted on the starting block 1.0 m above the water surface to prevent the connecting wire from interfering with the swimmer’s kicking motion. The setup for semi-tethered swimming used to obtain load-velocity profiles has been illustrated in Fig. 1b. The raw data were imported into Microsoft Excel for Windows 365 MSO (Microsoft Corp., Redmond, USA) for further processing and analysis of the load-velocity profile variables. The system-measured velocity (v) was adjusted for the angle of the cord relative to the horizontal plane according to the following Eq. (1)^10^.

where: v is the horizontal component of the velocity, v_abs_ is the absolute velocity measured by the software, 1.00 m is the height of the device above the water surface (the point where the cord is anchored), and L_c_ is the length of the cord (in meters) between the device and the swimmer.

Based on the linear regression analysis, a fitted trend line to these three points (3-point model) and two points (2-point model) representing the load-velocity profile has been obtained in the XY coordinate system against the corresponding load. The coefficient of determination (R²) was provided to assess the goodness of fit of the regression model^9^.

The steepness (Slope) of the regression line (-m ∙ s^−1^∙ kg^−1^) was calculated with the *SLOPE* () function using the existing XY values.

The point v_0_ (m ∙ s^−1^), at which a line will intersect the vertical Y-axis, represents the theoretical maximal velocity that each swimmer could achieve when the load at zero, and was obtained using the *INTERCEPT* () function from the existing XY data.

**Fig. 2:**
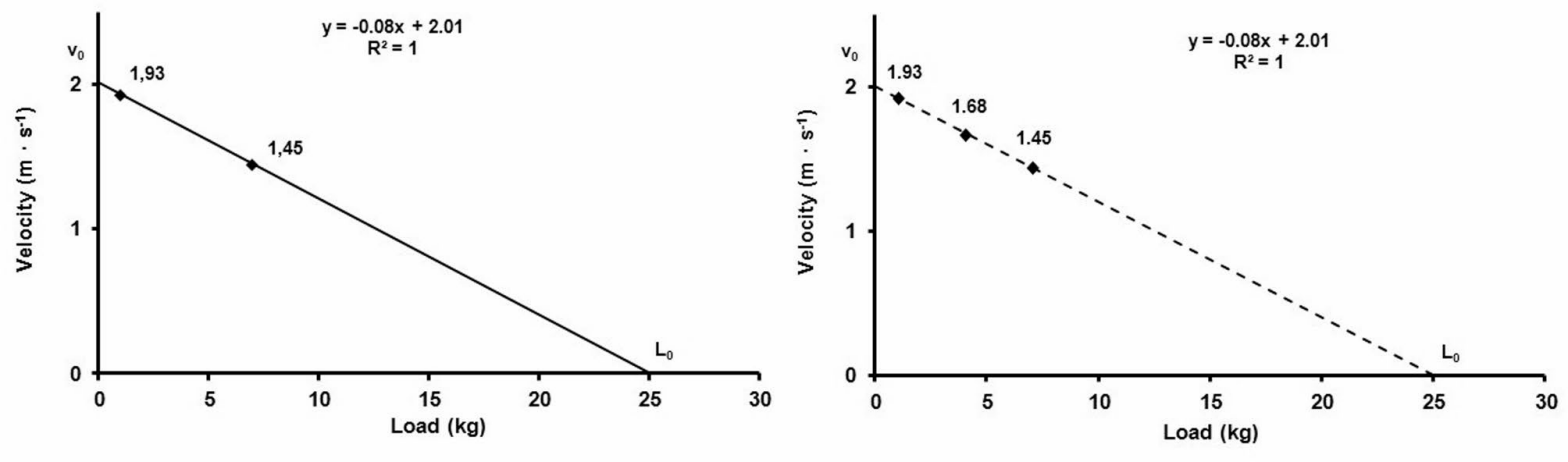
Example of load-velocity profile for FREE swimming condition in the 2-point (left) and 3-point (right) models for single male participants.

The point L_0_ (kg) at which a line will intersect the horizontal X-axis, which was the theoretical maximal load at which the velocity is zero that the swimmer can pull (without being towed backward) was calculated according to the following Eq. (2)^11^. The variables obtained from the L-v profiling in the 2-point and 3-point models have been presented in Fig. 2.

where: v_0_ is the theoretical maximal velocity with load at zero, and slope is the steepness of the regression line.

### Swimming performance

One day after the main session, participants completed two maximal-effort 25 m front crawl time trials. The start of the trials, as well as the standardized warm-up and rest intervals between them, were the same as in the main testing protocol. The time (s) to cover the 25 m distance was measured using an electronic start-up system, which consists of the Infinity Pro Start System with a speaker, the chronometer Quick Start Pro with display, and touchpads (Colorado Time, Loveland, USA). The principal researcher conducted timing, and the fastest time of the two trials was used for analysis.

### Statistical analysis

All statistical comparison analyses were performed using IBM SPSS Statistics software, version 26 (IBM Corp., Chicago, USA). Results are presented as mean ± standard deviation (x̄ ± SD) and 95% confidence intervals (95% CI). The normality of variable distributions was assessed using the Shapiro-Wilk test, and homogeneity of variances was evaluated using Levene’s test. A two-way ANOVA with main factors *model* (2-point, 3-point) and *conditions* (FREE, SNOR, ARDS) analyzed differences in the L-v relationship variables (v_0_, L_0_, slope). The validity of the 2-point method relative to the 3-point method for assessing the L-v relationship variables (v_0_, L_0_, slope) across three respiratory conditions was evaluated. A r-Pearson’s correlation coefficients (r) were calculated between L-v variables under the conditions and models to characterize the strength and direction of the relationship, as well as heteroscedasticity of the errors (i.e., relationship of the raw differences between the 2-point and 3-point methods with their average value). Furthermore, the correlation between 25 m freestyle performance and L-v variables indicators under the FREE swimming respiratory condition has been calculated. The strength of the correlation was interpreted according to the following thresholds: 0.1 (small), 0.3 (moderate), 0.5 (large), 0.7 (very large), and 0.9 (extremely large)^40^. The coefficient of determination (R²) was calculated to assess the proportion of variance explained between the 2-point and 3-point models, when no significant difference in velocity between L-v profile indicators between both models was observed. The Akaike information criterion (AIC) and the Bayesian information criterion (BIC) were reported to evaluate the goodness of fit of the 2-point regression model under three respiratory conditions. The smaller the AIC and the BIC values, the better the regression model^41^. Accordingly, the interclass correlation coefficient in mixed models (ICC_(2,1)_) and the standard error of estimate (SEE) were calculated. An overall bias was calculated and interpreted using the modified Cohen scale: trivial, < 0.20; small, 0.2–0.6; moderate, 0.6–1.2; 1.2–2.0.2.0, large; 2.0–4.0, very large; and > 4.0, extremely large^37^. To assess agreement between load-velocity variables across the three respiratory conditions, Bland-Altman plots were generated^42^. Limits of agreement (LoA) were used to compare the individual differences between variables, and mean differences ± 1.96 SD were provided for LoA lines. A p-value of ≤ 0.05 was considered statistically significant for all analyses. Results were presented according to the American Medical Association Manual of Style^43^.

## Results

The two-way ANOVA revealed no significant main effect in factor *condition* for v_0_ (F_(2,150)_ = 0.03, *p* = 0.967), L_0_ (F_(2,150)_ = 0.06, *p* = 0.942), slope (F_(2,150)_ = 0.01, *p* = 0.986), and also in factor *model* for v_0_ (F_(1,150)_ = 0.00, *p* = 0.947), L_0_ (F_(1,150)_ = 0.00, *p* = 0.986), and slope (F_(1,150)_ = 0.00, *p* = 1.00). There was no significant interaction between *condition* x *model* for load-velocity profile indicators (Table 2).

**Table 2:** Load-velocity profile indicators in 2-point and 3-point models for three respiratory conditions (mean ± SD) and 95% CI in the brackets.

Conditions	Model	v_0_ (m ∙ s^−1^)	L_0_ (kg)	Slope (-m ∙ s^−1^∙ kg^−1^)
**FREE**	3-point	1.80 ± 0.15 [1.74–1.86]	18.92 ± 5.89 [16.54–21.30]	−0.10 ± 0.03 [−0.11 - −0.09]
	2-point	1.80 ± 0.15 [1.74–1.86]	18.90 ± 5.90 [16.6–21.3]	−0.10 ± 0.03 [−0.11 - −0.09]
**SNOR**	3-point	1.80 ± 0.15 [1.73–1.86]	18.58 ± 4.52 [16.75–20.40]	−0.10 ± 0.02 [−0.11 - −0.09]
	2-point	1.80 ± 0.15 [1.73–1.86]	18.57 ± 4.51 [16.75–20.39]	−0.10 ± 0.02 [−0.11 - −0.09]
**ARDS**	3-point	1.79 ± 0.13 [1.74–1.84]	18.79 ± 5.41 [16.60–20.97]	−0.10 ± 0.02 [−0.11 - −0.09]
	2-point	1.79 ± 0.13 [1.74–1.84]	18.83 ± 5.38 [16.65–21.00]	−0.10 ± 0.02 [−0.11 - −0.09]

FREE *-* swimming without additional devices, SNOR *-* swimming with snorkel, ARDS *-* swimming with added respiratory dead space, v_0_ *-* estimated maximum velocity from the load-velocity profile, L_0_ *-* estimated maximum load from the load-velocity profile.

**Fig. 3:**
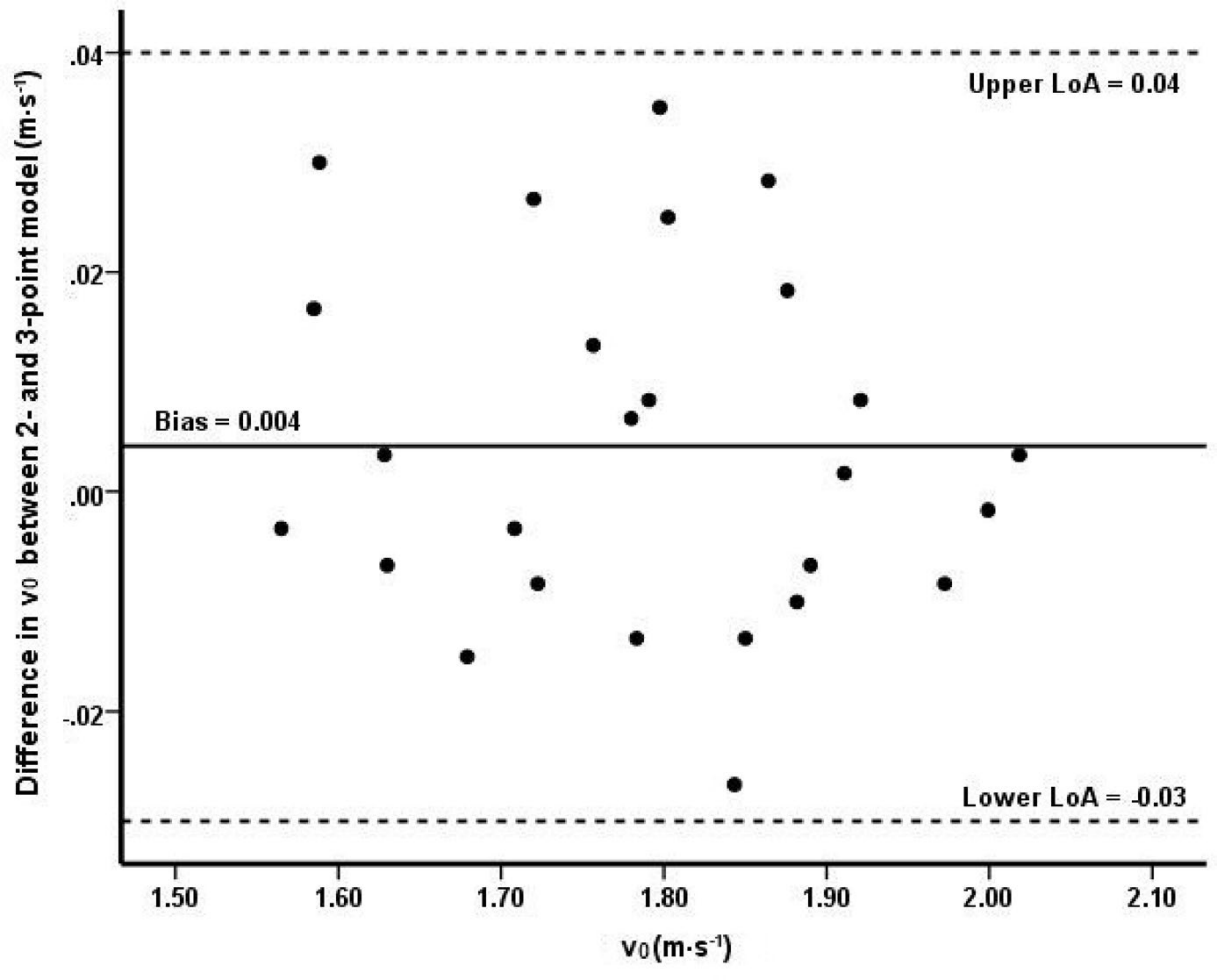
Example of a Bland-Altman plot showing the limits of agreement between the 2-point and 3-point models for the v_0_ variable in the ARDS condition.

Excellent agreement was observed between the 2- and 3-point models across all conditions for v_0_ and slope (*r* ≥ 0.99, ICC = 1.00, SEE ≤ 0.02), which corresponds to trivial bias (excluding v_0_ in ARDS, where bias was small). Also, excellent agreement was reported for L_0_ between both models (*r* = 1.00, ICC = 1.00), with slightly higher SEE equal to 0.11, 0.14. 0.18 for FREE, SNOR, and ARDS, respectively. In comparison to the 3-point model, the 2-point model showed only a trivial bias in L_0_ of 0.01 kg (95% CI: 0.03 to 0.05 kg) for FREE, −0.01 kg (95% CI: −0.06 to 0.05 kg) for SNOR, and a small bias of 0.04 kg (95% CI: −0.03 to 0.11 kg) for ARDS. A Bland-Altman analysis indicated that no individual values approached the limits of agreement. Thus, a plot was generated only as an example for the ARDS condition and the v_0_ variable. Measurement differences (y-axis) are plotted against the mean of the two measures (x-axis) for the analyzed variables. The solid horizontal line represents the mean difference (bias), and the two dotted horizontal lines indicate the upper and lower limits of agreement (± 1.96∙SD) (Fig. 3). The 2-point model accounted for more than 98% (R^2^ ≥ 0.98) of the variation across all L-v indicators in three respiratory conditions (Table 3). The AIC and BIC values obtained for comparisons between the 2- and 3-point models in each of the three respiratory conditions (FREE, SNOR, ARDS) indicated equivalent model fit (differences < 2 units), confirming the absence of meaningful differences between the models.

**Table 3:** Validity indicators: r-Pearson correlation coefficient (r), coefficient of determination (R^2^), interclass correlation coefficient (ICC_(2,1)_), standard error of Estimation (SEE), and cohen’s d between 2-point and 3-point models for L-v profile variables in the three conditions.

Parameters	Conditions	*r*	*R* ^2^	ICC_(2,1)_	SEE	Cohen’s d
v_0_ (m ∙ s^−1^)	FREE	1.00***	1.00	1.00	0.01	0.111
	SNOR	1.00***	0.99	1.00	0.01	0.107
	ARDS	0.99***	0.98	1.00	0.02	0.448
L_0_ (kg)	FREE	1.00***	1.00	1.00	0.11	0.147
	SNOR	1.00***	1.00	1.00	0.14	0.139
	ARDS	1.00***	1.00	1.00	0.18	0.408
Slope (-m ∙ s^−1^ ∙ kg^−1^)	FREE	1.00***	1.00	1.00	0.00	0.000
	SNOR	1.00***	1.00	1.00	0.00	0.000
	ARDS	1.00***	1.00	1.00	0.00	0.000

FREE *-* swimming without additional devices, SNOR *-* swimming with snorkel, ARDS *-* swimming with added respiratory dead space, v_0_ *-* estimated maximum velocity from the load-velocity profile, L_0_ *-* estimated maximum load from the load-velocity profile. Statistically significant: *** *- p* < 0.001.

To examine the relevance of L-v profile parameters for swim sprint performance, Pearson’s correlation coefficients were calculated separately for the 3-point and 2-point models (Tables 4 and 5). In both models, strong to very strong correlations were found between maximal-effort 25 m front crawl time trials (14.66 ± 1.34 s) and the L-v profile parameters. The strongest associations were observed for v_0_ (*r* = −0.82 to −0.84), followed by L_0_ (*r* = −0.72). Negative correlations indicate that higher theoretical maximal velocity and load are associated with faster 25 m performance. Correlations for slope (*r* = −0.58) were weaker. The strength and pattern of correlations were comparable between models, supporting the validity of the 2-point approach and practical usefulness.

**Table 4:** r-Pearson correlation coefficient between 25 m swimming performance and load-velocity variables in the 3-point model for FREE swimming condition.

Parameters	t_25m_ (s)	v_0_ (m ∙ s^−1^)	L_0_ (kg)	Slope (-m ∙ s^−1^∙ kg^−1^)
t_25m_ (s)	x			
v_0_ (m ∙ s^−1^)	−0.82***	x		
L_0_ (kg)	−0.72***	0.48*	x	
Slope (-m ∙ s^−1^∙ kg^−1^)	−0.58**	0.28	0.92***	x

t_25m_ - time for the 25 m front crawl, v_0_ *-* estimated maximum velocity from the load-velocity profile, L_0_ *-* estimated maximum load from the load-velocity profile. Statistically significant: * - *p* < 0.05, ** - *p* < 0.01, *** - *p* < 0.001.

**Table 5:** r-Pearson correlation coefficient between 25 m swimming performance and load-velocity variables in the 2-point model for FREE swimming condition.

Parameters	t_25m_ (s)	v_0_ (m ∙ s^−1^)	L_0_ (kg)	Slope (-m ∙ s^−1^∙ kg^−1^)
t_25m_ (s)	x			
v_0_ (m ∙ s^−1^)	−0.84***	x		
L_0_ (kg)	−0.72***	0.49*	x	
Slope (-m ∙ s^−1^∙ kg^−1^)	−0.58**	0.30	0.92***	x

t25m - time for the 25 m front crawl, v_0_ - estimated maximum velocity from the load-velocity profile, L_0_ - estimated maximum load from the load-velocity profile. Statistically significant: * - *p* < 0.05, ** - *p* < 0.01, *** - *p* < 0.001.

## Discussion

This is the first study to examine the agreement between the 2- and 3-point models in estimating load-velocity profile parameters in three respiratory conditions, free breathing, snorkel, and added respiratory dead space mask in swimmers. The principal finding of this study was that a 2-point model can be used to estimate L-v parameters in front crawl swimming as proficiently as a 3-point model in different respiratory conditions. Secondly, the L-v profile parameters from the 2-point model are strongly to very strongly related with 25 m sprint crawl performance.

The 2-point model was thoroughly examined in relation to the 3-point model. Validity indicators showed extremely large (almost perfect) correlations (*r* ≥ 0.99) between L-v profile variables. The use of the 2-point method is also justified by the R^2^ values of the L-v profiles, which were very well fitted by linear regressions (range: 0.98–1.00) and were consistent with previous findings. In a study where swimmers performed 12 trials pulling different loads, a linear relationship between load and velocity was reported (R^2^ = 0.98)^9^. Notably, high R^2^ values were reported for the L-v profile using three and four trials of butterfly (0.98 and 0.97, respectively)^3^, and three and five trials of front crawl (0.99 for all conditions)^10^. Moreover, for three trials with external load in backstroke^13^ and breaststroke^14^, the R^2^ value was 0.99. The results presented in this study indicate that the high R^2^ values confirm a linear relationship between load and velocity in semi-tethered freestyle swimming, accounting for more than 98% of the variation across all conditions. L-v relationships determined from 2-point and 3-point resisted trials across all conditions were highly comparable for L_0_, v_0_, slope, and showed excellent agreement with trivial and bias. Additionally, ICC were similar to those reported for three (range: 0.90–0.98) and five (range: 0.92–0.98) resisted trials in freestyle swimming^10^. Similarly, during resisted running sprints, the 5-point method and two variants of the 2-point method were compared. Regardless of the 2-point method variant and L-v relationship variable, correlations with the multiple-point method were positive and nearly perfect (*r* > 0.90), errors were homoscedastic (r^2^ < 0.10), and neither fixed nor proportional bias was observed. Trivial, non-statistically significant differences were found for both L_0_ and v_0_ variables^32^. The importance and relevance of the 2-point model across different exercise modalities were also highlighted by reliability analyses. For example, during the Smith machine bench pull exercise, both the standard 6-point and 2-point methods provided greater reliability for all L-v relationship variables compared to the modified multiple-point method, in which the data point that most reduced the coefficient of determination was omitted from the L-v modeling. In turn, the 2-point method demonstrated greater reliability than the standard 6-point method^31^. However, reliability analysis was not performed in the present study; therefore, further research should evaluate the 2-point model’s reliability under swimming conditions.

The two-way ANOVA revealed no significant differences in the L-v profile indicators between respiratory conditions (FREE, SNOR, ARDS), regardless of whether a 2- or 3-point model was applied (*p* > 0.05). This suggests that the use of a swim snorkel or ARDS mask did not meaningfully affect L-v profile parameters. Recently, the use of swimming aids, such as a snorkel has been analyzed to evaluate its applicability during a training intervention study^44,45^, symmetry of arm coordination in front crawl^46^ and use of single tube snorkel for respiratory data acquisition during swimming^47^. Furthermore, there were several studies where the snorkel affected changes in the biomechanical parameters in the front crawl and breaststroke^18^. In turn, in other studies, using a snorkel did not cause any changes in stroke rate and stroke length during 5 min of front-crawl swimming in a flume^48^. The use of sprint training equipment in competitive swimming is not a recent practice. Devices such as swim snorkels with different characteristics are frequently used in workouts by swimmers of all training levels and age groups, as well as during competitions, e.g., surface fin swimming. Thus, these findings indicate that center-mount swim snorkels differ from other swimming tools, such as fins^4^ or respiratory gas analyzers^18^. Snorkels can be applied in diagnostics, maximal sprint training, and data collection by researchers without inducing biomechanical disturbances that could reduce swimming velocity while simultaneously providing stimulation. However, direct comparisons should be evaluated in future investigations.

All L-v parameters were significantly related to a 25 m front crawl performance. For both the 3- and 2-point models, Pearson’s correlation coefficients indicated, respectively, very large and large correlations between t_25_ and v_0_ (*r* = −0.82 to −0.84), L_0_ (*r* = −0.72), and slope (*r* = −0.58). This suggests that L-v profiling is an excellent method for predicting short-distance front crawl swimming performance and velocity. The previous investigation among male national-level swimmers also demonstrated that L-v profiling is a useful method for assessing sprint performance across all four swimming strokes. Significant correlations were found between 50 m butterfly race time and v_0_ (*r* = −0.80), L_0_ (*r* = −0.62), and slope as v_0_-v_race_ (*r* = 0.56)^3^. Similarly, significant correlations were observed between 50 m front crawl race time and v_0_ (*r* = −0.67), L_0_ (*r* = −0.55), and slope (*r* = −0.46)^11^. Additionally, significant correlations were reported between 50 m backstroke race time and v_0_ (*r* = −0.70), L_0_ (*r* = −0.72), and slope (*r* = −0.63)^13^. Finally, in the 50 m breaststroke, significant correlations were found between race time and v_0_ (*r* = −0.51), L_0_ (*r* = −0.61), and slope (*r* = −0.28)^14^. From the perspective that v_0_ represents the theoretical maximum velocity, it is logical that it is largely negatively correlated with t_25_. Similarly, the relationship between L_0_ and t_25_ can be explained by the generation of maximum force during tethered swimming, which was correlated with actual sprint swimming performance at 50 m (*r* = −0.82)^49^. A large, significant correlation between L_0_ and slope (*r* = −0.92), along with a moderate, non-significant correlation between v_0_ (*r* = −0.28 to −0.30) and slope, was consistent with a large, significant association between slope and t_25_ (*r* = −0.58). This aligns with L-v studies on butterfly, freestyle, backstroke, and breaststroke, in which the authors reported that slope showed a moderate-to-large association influenced by L_0_. This suggests that the relationship between slope and swimming time can also be explained by the influence of L_0_ (respectively to the strokes described − 0.36, 0.95, 0.97, 0.93)^3,11,13,14^. A moderate and significant correlation between L_0_ and _V0_ was observed (*r* = 0.48 to 0.49), likely reflecting that swimmers simultaneously maximized propulsive force production and minimized resistive forces to achieve high velocity. Similarly, in L-v profiling, strong correlations were reported for backstroke (*r* = 0.70) and breaststroke (*r* = 0.97)^13,14^. However, in butterfly and front crawl, some swimmers employed different strategies, as indicated by the non-significant correlations between L_0_ and v_0_ (*r* = 0.47 and *r* = 0.33, respectively). Some swimmers relied primarily on propulsive force production, whereas others focused on minimizing resistive forces to achieve a high v_0_. In high-loading conditions, swimmers do not focus on minimizing resistive forces but instead concentrate on producing large propulsive forces. However, under low-loading conditions, avoiding drag becomes important due to high forward velocity^11^. Literally, a high v_0_ with a steep slope means that the swimmer might not have produced a large propulsive force but may have used a technique to minimize resistive force. Hence, the slope parameters can be considered indicators of resistive force, because the steeper the slope, the greater the ability to minimize resistance.

The 2-point approach to L-v profiling has the potential to provide swimmers with a simple yet accurate method to more individualized monitoring and training of strength (propulsion) and technical capabilities (resistance reduction). Performing fewer sprint repetitions across several loads can save time and reduce fatigue. For instance, if an athlete is unable to show their maximum swimming speed during a heavy load trial because of fatigue, the resulting load-velocity slope becomes artificially steeper, leading to an overestimated v_0_ value^3^. As a result, practitioners may be more willing to prescribe maximal sprinting, due to the associated lower strain and reduced risk of injury. The 2-point method can be easily implemented regularly, as it is based on common, swim-specific movements (semi-tethered swimming), and can therefore be used for long-term monitoring and training processes. A strong correlation between the 25 m front crawl time trial and the L-v parameters (v_0_, L_0_, and slope) suggests that L-v variables are reliable predictors of swim sprint performance. This information can be used to develop personalized training programs. It also provides a useful method for comparing swimmers and monitoring the long-term development of free-swimming velocity in front crawl performance. Finally, the 2-point method can be applied under respiratory conditions involving the use of a swim snorkel or an ARDS mask, which are employed in training to improve technique and to develop anaerobic capacity during maximal-effort time trials. Therefore, the results are encouraging for coaches to adopt the 2-point method as an accurate, quick, and relatively fatigue-free approach to estimating the L-v profile for monitoring swimmers’ performance. This study also contributes to the level of knowledge available in the literature related to L-v profiling in swimming and can be a support for those who want to further study the topic.

There are some limitations to the present study that should be considered when interpreting the results. Firstly, individual differences (gender, training level, technique) can significantly alter the shape of the profile and its practical applicability^50^. This study examined male swimmers. Thus, the findings may not be fully generalizable to other cohorts. Secondly, participants represented the lowest level of athletic performance. However, high-level athletes, including sprinters and distance swimmers, typically possess different proportions of muscle fiber types^51^, which may influence profiling outcomes. Thirdly, the study did not provide stroke cycle parameters like stroke frequency as well as stroke length data, which could help identify changes in swimming technique resulting from the use of different resistance levels across different respiratory conditions. Fourthly, the analysis was limited only to the front crawl technique. However, it is the fastest swimming stroke and therefore the most appropriate for evaluating human swimming performance. Fifthly, the time obtained in a 25 m sprint trial may not accurately represent a swimmer’s performance in a 50 m race in competition^52^. Sixthly, assigning individual loads is more robust and has less variance than using fixed loads when assessing swimmers with different body masses, thereby retaining a more stable stroke pattern^27^. Thus, the methodology with individual loads should be tested. Finally, the validation of the 2-point method against the 3-point method was presented. However, measurements should be repeated under the same conditions to determine its reliability. In future studies, it would be particularly interesting to address and overcome the limitations identified in the present research.

## Conclusions

The 2-point model, which involves semi-tethered sprints performed at maximal effort with two resistance loads (1 and 7 kg), allows for the assessment of load-velocity profile parameters (L_0_, v_0_, slope) as reliably as the 3-point model (1, 4, and 7 kg). However, it offers a more practical, less physically demanding, time-efficient, and suitable method for coaches and researchers to assess performance in swimmers.

Additionally, the swim snorkel and the ARDS mask did not affect changes in the L-v profile during swim sprint performance. Thus, both types of swim tools can be effectively used in swim training to enhance velocity.

Furthermore, in both models (2- and 3-point), strong to very strong relationships have been observed between all L-v profile parameters and a 25 m front crawl time trial. This indicates that the L-v profile is a useful method for predicting and assessing velocity capabilities in 25 m front crawl performance.

## Data Availability

The data cannot be shared publicly due to concerns regarding the inadvertent disclosure of personal health information and performance data of athletes. Data are available from the Wroclaw University of Health and Sport Sciences Research Ethics Committee (contact: katedra.oiz@awf.wroc.pl) or from the first author (contact: stefan.szczepan@awf.wroc.pl) for researchers who meet the criteria for access to confidential data. Access to the dataset requires conducting a joint investigation with the research staff.

## References

[1] Cahill, M. J. et al. Sled-pull load-velocity profiling and implications for sprint training prescription in young male athletes. *Sports (Basel)*. **7**, 119. 10.3390/sports7050119 (2019). 31137511 10.3390/sports7050119 PMC6572326

[2] Jiménez-Reyes, P. et al. Changes in mechanical properties of sprinting during repeated sprint in elite rugby sevens athletes. *Eur. J. Sport Sci.* **19**, 585–594. 10.1080/17461391.2018.1542032 (2019). 30409072 10.1080/17461391.2018.1542032

[3] Gonjo, T., Eriksrud, O., Papoutsis, F. & Olstad, B. H. Relationships between a load-velocity profile and sprint performance in butterfly swimming. *Int. J. Sports Med.* **41** (7), 461–467. 10.1055/a-1103-2114 (2020). 32059244 10.1055/a-1103-2114

[4] Wang, S., Zhao, Y., Chen, X. & Shen, Y. Effect of increasing the foot area on the Load-Velocity relationship of the underwater Dolphin kick. *J. Hum. Kinet*. **95**, 17–27. 10.5114/jhk/189796 (2025). 39944981 10.5114/jhk/189796 PMC11812160

[5] Hollander, A. P. et al. Measurement of active drag during crawl arm stroke swimming. *J. Sports Sci.* **4**, 21–30. 10.1080/02640418608732094 (1986). 3735480 10.1080/02640418608732094

[6] Kolmogorov, S. V. & Duplishcheva, O. A. Active drag, useful mechanical power output and hydrodynamic force coefficient in different swimming strokes at maximal velocity. *J. Biomech.* **25**, 311–318. 10.1016/0021-9290(92)90028-Y (1992). 1564064 10.1016/0021-9290(92)90028-y

[7] Formosa, D. P., Toussaint, H. M., Mason, B. R. & Burkett, B. Comparative analysis of active drag using the MAD system and an assisted towing method in front crawl swimming. *J. Appl. Biomech.* **28**, 746–750 (2012). 22695220 10.1123/jab.28.6.746

[8] Samson, M., Monnet, T., Bernard, A., Lacouture, P. & David, L. Analysis of a swimmer’s hand and forearm in impulsive start from rest using computational fluid dynamics in unsteady flow conditions. *J. Biomech.* **67**, 157–165. 10.1016/j.jbiomech.2017.12.003 (2018). 29269003 10.1016/j.jbiomech.2017.12.003

[9] Dominguez-Castells, R. & Arellano, R. Effect of different loads on stroke and coordination parameters during freestyle semi-tethered swimming. *J. Hum. Kinet*. **32**, 33–41. 10.2478/v10078-012-0021-9 (2012). 23486657 10.2478/v10078-012-0021-9 PMC3590860

[10] Olstad, B. H., Gonjo, T., Njøs, N., Abächerli, K., Eriksrud, O. & Reliability of load-velocity profiling in front crawl swimming. *Front. Physiol.* **11**, 574306. 10.3389/fphys.2020.574306 (2020). 33071829 10.3389/fphys.2020.574306 PMC7538691

[11] Gonjo, T., Njøs, N., Eriksrud, O. & Olstad, B. H. The relationship between selected load-velocity profile parameters and 50 m front crawl swimming performance. *Front. Physiol.* **12**, 625411. 10.3389/fphys.2021.625411 (2021). 33679439 10.3389/fphys.2021.625411 PMC7933527

[12] Szczepan, S. et al. Reliability of a semi-tethered front crawl sprint performance test in adolescent swimmers. *Front. Physiol.* **14**, 1260346. 10.3389/fphys.2023.1260346 (2023). 38156067 10.3389/fphys.2023.1260346 PMC10753824

[13] Olstad, B. H., Ljødal, I., Karlsson, R. & Gonjo, T. The relationship between backstroke swimming sprint performance and load-velocity profiles. *ISBS Proc. Arch.* **40(1)**, 128 (2022).

[14] Olstad, B. H., Hunger, L., Ljødal, I., Ringhof, S., Gonjo, T. & The relationship between load-velocity profiles and 50 m breaststroke performance in national-level male swimmers. *J. Sports Sci.* **42** (16), 1512–1518. 10.1080/02640414.2024.2397234 (2024). 39231296 10.1080/02640414.2024.2397234

[15] Toussaint, H. & Truijens, M. Biomechanical aspects of peak performance in human swimming. *Anim. Biol.* **55** (1), 17–40 (2005).

[16] Marinho, D. et al. The hydrodynamic drag during gliding in swimming. *J. Appl. Biomech.* **25** (3), 253–257 (2009). 19827475 10.1123/jab.25.3.253

[17] Maglischo, E. W. *Swimming Fastest* (Human Kinetics, 2003).

[18] Barbosa, T. et al. Kinematical changes in swimming front crawl and breaststroke with the AquaTrainer^®^ snorkel. *Eur. J. Appl. Physiol.* **109**, 1155–1162. 10.1007/s00421-010-1459-x (2010). 20379828 10.1007/s00421-010-1459-x

[19] Szczepan, S., Michalik, K., Borkowski, J. & Zatoń, K. Effects of swimming with added respiratory dead space on cardiorespiratory fitness and lipid metabolism. *J. Sports Sci. Med.* **19** (1), 95–101 (2020). 32132832 PMC7039034

[20] Danek, N., Szczepan, S., Wróblewska, Z., Michalik, K. & Zatoń, M. Hypercapnic warm-up and re-warm-up–a novel experimental approach in swimming sprint. *PLoS ONE*. **20** (1), e0314089. 10.1371/journal.pone.0314089 (2025). 39879158 10.1371/journal.pone.0314089 PMC11778800

[21] Smolka, L., Borkowski, J. & Zatoń, M. The effect of additional dead space on respiratory exchange ratio and carbon dioxide production due to training. *J. Sports Sci. Med.* **13** (1), 36–43 (2014). 24570603 PMC3918565

[22] Michalik, K., Zalewski, I., Zatoń, M., Danek, N. & Bugajski, A. High intensity interval training with added dead space and physical performance of amateur triathletes. *Med. Sport*. **4** (4), 247–255. 10.5604/01.3001.0012.9668 (2018).

[23] Szczepan, S., Danek, N., Michalik, K., Wróblewska, Z. & Zatoń, K. Influence of a six-week swimming training with added respiratory dead space on respiratory muscle strength and pulmonary function in recreational swimmers. *Int. J. Environ. Res. Public. Health*. **17** (16), 5743. 10.3390/ijerph17165743 (2020). 32784446 10.3390/ijerph17165743 PMC7459907

[24] Szczepan, S., Michalik, K. & Hebisz, R. Does a six-week intervention with added respiratory dead space volume in swimming improve haematological and immunological status? Balt. *J. Health Phys. Act.* **14** (4).), Article 6 (10.29359/BJHPA.14.4.06 (2022).

[25] Adam, J., Zatoń, M. & Damska-Wierzbicka, I. Physiological adaptation to high intensity interval training with added volume of respiratory dead space in club swimmers. *Med. Sport*. **4** (4), 223–237. 10.5604/1232406X.1194805 (2015).

[26] Petrakos, G., Morin, J. B. & Egan, B. Resisted sled sprint training to improve sprint performance: A systematic review. *Sports Med.* **46** (3), 381–400. 10.1007/s40279-015-0422-8 (2016). 26553497 10.1007/s40279-015-0422-8

[27] Olstad, B. H., Ljødal, I. & Gonjo, T. Variance in front crawl load-velocity testing using fixed or individual loads. In: Witt, M. (ed.), XIVth Int. Symp. Biomech. Med. Swimming Proc. 1Leipzig, Germany, (2023).

[28] Driss, T. & Vandewalle, H. The measurement of maximal (anaerobic) power output on a cycle ergometer: a critical review. *Biomed. Res. Int.* **2013** (589361). 10.1155/2013/589361 (2013). 10.1155/2013/589361 PMC3773392 24073413

[29] Jaric, S. Two-load method for distinguishing between muscle force, velocity, and power-producing capacities. *Sports Med.* **46** (11), 1585–1589. 10.1007/s40279-016-0531-z (2016). 27075326 10.1007/s40279-016-0531-z PMC5056118

[30] García-Ramos, A. et al. Feasibility of the 2-Point method for determining the 1-Repetition maximum in the bench press exercise. *Int. J. Sports Physiol. Perform.* **13** (4), 474–481. 10.1123/ijspp.2017-0374 (2018). 28872384 10.1123/ijspp.2017-0374

[31] Miras-Moreno, S., García-Ramos, A., Jukic, I. & Pérez-Castilla, A. Two-point method applied in field conditions: A feasible approach to assess the load-velocity relationship variables during the bench pull exercise. *J. Strength. Cond Res.* **37** (7), 1367–1374. 10.1519/JSC.0000000000004405 (2023). 36728020 10.1519/JSC.0000000000004405

[32] Jiménez-Reyes, P. et al. The 2-point method for a simplified individualization of resisted sprint assessment and training. *OSF Preprint*. 10.31219/osf.io/8vq73_v1 (2025).

[33] García-Ramos, A. The 2-point method: theoretical basis, methodological considerations, experimental support, and its application under field conditions. *Int. J. Sports Physiol. Perform.* **18** (10), 1092–1100. 10.1123/ijspp.2023-0127 (2023). 37541677 10.1123/ijspp.2023-0127

[34] Ruiz-Navarro, J. J., López-Belmonte, Ó., Gay, A., Cuenca-Fernandez, F. & Arellano, R. A new model of performance classification to standardize the research results in swimming. *Eur. J. Sport Sci.* **23** (4), 478–488. 10.1080/17461391.2022.2046174 (2022). 35193458 10.1080/17461391.2022.2046174

[35] Fletcher, G. F. et al. Exercise standards for testing and training: a scientific statement from the American heart association. *Circulation* **128** (8), 873–934. 10.1161/CIR.0b013e31829b5b44 (2013). 23877260 10.1161/CIR.0b013e31829b5b44

[36] Faul, F., Erdfelder, E., Lang, A. G. & Buchner, A. G*Power 3: a flexible statistical power analysis program for the social, behavioral, and biomedical sciences. *Behav. Res. Methods*. **39**, 175–191. 10.3758/bf03193146 (2007). 17695343 10.3758/bf03193146

[37] Levine, T. R. & Hullett, C. R. Eta squared, partial Eta squared, and misreporting of effect size in communication research. *Hum. Commun. Res.* **28**, 612–625. 10.1111/j.1468-2958.2002.tb00828.x (2002).

[38] Neiva, H. P., Marques, M. C., Barbosa, T. M., Izquierdo, M. & Marinho, D. A. Warm-up and performance in competitive swimming. *Sports Med.* **44** (3), 319–330. 10.1007/s40279-013-0117-y (2014). 24178508 10.1007/s40279-013-0117-y

[39] Hancock, A. P., Sparks, K. E. & Kullman, E. L. Postactivation potentiation enhances swim performance in collegiate swimmers. *J. Strength. Cond Res.* **29** (4), 912–917. 10.1519/JSC.0000000000000744 (2015). 25426510 10.1519/JSC.0000000000000744

[40] Hopkins, W. G., Marshall, S. W., Batterham, A. M. & Hanin, J. Progressive statistics for studies in sports medicine and exercise science. *Med. Sci. Sports Exerc.* **41**, 3–13. 10.1249/MSS.0b013e31818cb278 (2009). 19092709 10.1249/MSS.0b013e31818cb278

[41] Bozdogan, H. Model selection and akaike’s information criterion (AIC): the general theory and its analytical extensions. *Psychometrika* **52** (3), 345–370. 10.1007/BF02294361 (1987).

[42] Bland, J. M. & Altman, D. G. Statistical methods for assessing agreement between two methods of clinical measurement. *Lancet* **1** (8476), 307–310 (1986). 2868172

[43] AMA Manual of Style. Committee AMAMoS. [Internet]. Feb 03 [cited 2025 Mar 01]. (2020). Available from: https://academic.oup.com/amamanualofstyle

[44] Pinna, M. et al. Assessment of the specificity of cardiopulmonary response during tethered swimming using a new snorkel device. *J. Physiol. Sci.* **63** (1), 7–16. 10.1007/s12576-012-0226-7 (2013). 22915172 10.1007/s12576-012-0226-7 PMC10717671

[45] Zeller, S. et al. Influence of a newly developed swim snorkel: An intervention study. In: Ferrauti, A. (eds.)Bochum, Germany, Book of Abstracts, 22nd Annu. Congr. Eur. Coll. Sport Sci. 113 (2017).

[46] Seifert, L., Chehensse, A., Tourny-Chollet, C., Lemaitre, F. & Chollet, D. Effect of breathing pattern on arm coordination symmetry in front crawl. *J. Strength. Cond Res.* **22** (5), 1670–1676. 10.1519/JSC.0b013e318182029d (2008). 18714216 10.1519/JSC.0b013e318182029d

[47] Fernandes, R. J., Figueiredo, P. & Vilas-Boas, J. P. About the use and conclusions extracted from a single tube snorkel used for respiratory data acquisition during swimming. *J. Physiol. Sci.* **63** (2), 155–157. 10.1007/s12576-012-0249-0 (2013). 23292732 10.1007/s12576-012-0249-0 PMC10716952

[48] Ruiz-Teba, A., Arellano, R. & López-Contreras, G. Technical and physiological responses of swimming crawl stroke using hand paddles, fins and snorkel in swimming flume: a pilot study. In: Colloud, F., Domalain, M. & Monnet, T. (eds.) 33rd Int. Conf. Biomech. Sports, 695–698Poitiers, France, (2015).

[49] Loturco, I. et al. A correlational analysis of tethered swimming, swim sprint performance and dry-land power assessments. *Int. J. Sports Med.* **37** (3), 211–218. 10.1055/s-0035-1559694 (2016). 26669251 10.1055/s-0035-1559694

[50] Weakley, J. et al. Velocity-based training: from theory to application. *Strength. Cond J.* **43** (2), 31–49. 10.1519/SSC.0000000000000560 (2021).

[51] Gerard, E. S., Caiozzo, V. J., Rubin, B. D., Prietto, C. A. & Davidson, D. M. Skeletal muscle profiles among elite long, middle, and short distance swimmers. *Am. J. Sports Med.* **14** (1), 77–82. 10.1177/036354658601400113 (1986). 3752351 10.1177/036354658601400113

[52] Vantorre, J., Seifert, L., Fernandes, R. J., Boas, V., Chollet, D. & J.P. & Comparison of grab start between elite and trained swimmers. *Int. J. Sports Med.* **31**, 887–893. 10.1055/s-0030-1265150 (2010). 20862626 10.1055/s-0030-1265150

